# Reaction of pharmacological active tris-(2-hydroxyethyl)ammonium 4-chlorophenylsulfanylacetate with ZnCl_2_ or NiCl_2_: first conversion of a protic ionic liquid into metallated ionic liquid

**DOI:** 10.1186/1752-153X-7-34

**Published:** 2013-02-19

**Authors:** Anna N Mirskova, Sergey N Adamovich, Rudolf G Mirskov, Uwe Schilde

**Affiliations:** 1 A. E. Favorsky Irkutsk Institute of Chemistry, Siberian Branch, Russian Academy of Sciences, 1 Favorsky Str, 664033, Irkutsk, Russia; 2 University of Potsdam, Institute of Chemistry, Karl-Liebknecht-Str. 24-25, D-14476 Potsdam, Germany

## Abstract

The reaction of pharmacological active protic ionic liquid tris-(2-hydroxyethyl)ammonium 4-chlorophenylsulfanylacetate H^+^N(CH_2_CH_2_OH)_3_ ∙ (^-^OOCCH_2_SC_6_H_4_Cl-4) (**1**) with zinc or nickel chloride in a ratio of 2:1 affords stable at room temperature powder-like adducts [H^+^N(CH_2_CH_2_OH)_3_]_2_ ∙ [M(OOCCH_2_SC_6_H_4_Cl-4)_2_Cl_2_]^2-^, M = Zn (**2**), Ni (**3**). By recrystallization from aqueous alcohol compound **2** unexpectedly gives Zn(OOCCH_2_SC_6_H_4_Cl-4)_2_ ∙ 2H_2_O (**4**). Unlike **2**, compound **3** gives crystals [N(CH_2_CH_2_OH)_3_]_2_Ni^2+^ · [^-^OOCCH_2_SC_6_H_4_Cl-4]_2_ (**5**), which have a structure of metallated ionic liquid. The structure of **5** has been proved by X-ray diffraction analysis. It is the first example of the conversion of a protic ionic liquid into potentially biological active metallated ionic liquid (**1** → **3** → **5**).

## Findings

Alkanolammonium salts of inorganic and carbonic acids, also known as protic ionic liquids (PILs), have been the subject of many studies [1,2]. Depending on cation and anion structure, PILs can be liquid (room temperature ionic liquids) [2] or solid compounds with m.p. up to 100°С and even higher (176°С [3]). For example, 2-hydroxyethylammonium nitrate, H_3_^+^NCH_2_CH_2_OH ∙ NO_3_^-^, synthesized in 1888, has m.p. 52°С [4]. At the same time, 2-hydroxy-ethylammonium formiate, H_3_^+^NCH_2_CH_2_OH ∙ ^-^OOCН, represents a typical room temperature PIL with extremely low freezing point (−82°С) [5]. Alkanolammonium PILs are used as catalysts in chemical reactions, as electrolytes in full cells, gas (such as CO_2_ and SO_2_) solvents and crystaline cellulose solvents, for desulfurization of fuel, enzymes stabilizers and promoters of their activity and for protein purification [6-10]. Also, they are employed for the design of nano-structured compounds [11]. Their toxicity and biological degradation have been studied [12,13].

Among the objects of our previous investigations were PILs containing cations of biologically active 2-hydroxyethylamines and anions of aroxy- and aryl(heteryl)sulfanyl(sulfonyl)acetic acids R_1_R_2_N^+^H(CH_2_CH_2_OH)_3-n_ ∙ (^-^OOCCH_2_XR), R = Ar, Het; R_1_, R_2_ = H, Alk; X = O, S, SO_2_; n = 0–2.

These PILs are air-stable solids (m.p. 37-95^о^С) or viscous liquids, well soluble in water and polar solvents, representing a new class of pharmacologically active substances. Showing low toxicity (LD_50_ = 1500–6000 mg/kg), they possess antiaggregation, antithrombotic, membrane-stabilizing, antioxidant, antisclerotic, adaptogenic, analgesic, cardiotropic, hypocholesterolemic, hemo- and immunotropic activities. These PILs protect the mammalians and humans from shock, toxic stress, alcohol and heavy metal intoxication, and radiation. Their antitumor activity considerably exceeds or differs from the effect of the initial biologically active acids and alkanolamine [14-19].

They also exert pronounced growth-stimulating activity at very low concentrations (10^–4^ - 10^–10^ wt %) toward beneficial bacteria, yeasts, and fungi used in large-scale biotechnology processes (white biotechnology [20]) for manufacture of fodder, baker’s yeasts and citric acid, barley sprouting for the preparation of brewer’s malt, and breeding of silkworms [21].

Recently we have shown that metallated ionic liquid tris-(2-hydroxyethyl)amine-bis-(2-methylphenoxyacetate)zinc N(CН_2_СН_2_ОН)_3_Zn^2+^ · 2(^-^ООССН_2_OC_6_H_4_-Me-2) exhibits a pronounced anti-sclerotic effect [22].

We have assumed that the incorporation of essential metals (so-called “metals of life”), which are of vital importance for all living organisms: Ca, Mg, Zn, Mn, Cu, Fe, Co, Ni, etc. [23,24], can enhance or alter the biological activity of protic alkanolammonium ionic liquids.

To reach this goal, in this work we have studied the reaction of PIL tris-(2-hydroxyethyl)-ammonium 4-chlorophenylsulfanylacetate **(1)** (a non-toxic compound possessing antithrombotic, antioxidant and immunotropic activity) with Zn and Ni chlorides.

**Scheme 1:**
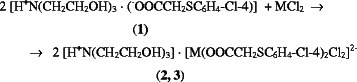
Synthesis of compounds 2 (M = Zn) and 3 (M = Ni).

The interaction of **1** with metal salts furnishes powder compounds **2** and **3** (Scheme  1).

According to the data of IR spectroscopy, the compounds **2** and **3** contain coordination bonds HО···M with the ОН groups of two molecules of protonated triethanolamine and coordination bonds M-О with two carboxylate anions of 4-chlorophenylsulfanylacetic acid. So, the IR spectrum of **3** shows the absorption bands ν(Ni-ОС) 396 cm^-1^, ν_as_(COO^−^) and ν_s_(COO^−^) at 1583 and 1401 cm^-1^, Δν = ν_as_(COO^−^) - ν_s_(COO^−^) = 182 cm^-1^, characterizing bidentate coordination bonds of nickel atom with carboxylate anions; absorption bands typical for protonated triethanolamine HN^+^(CH_2_CH_2_OH)_3_ at 548, 549 and 397 cm^-1^, ν(N^+^H) is a broad band at 2500–2700 cm^-1^; absorption band of the OH group at 3312 cm^-1^.

**Scheme 2:**
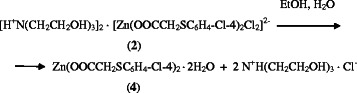
Conversion of compound 2 into compound 4.

Powders **2** and **3** are stable at room temperature. However, on storage in solutions of organic solvents they change their composition and structure. So, for example, when recrystallized from aqueous alcohol (75°C), the powder adduct **2** is unexpectedly converted into zinc di-(4-chloro-phenylsulfonyl) acetate dihydrate **4** (Scheme  2).

**Scheme 3:**
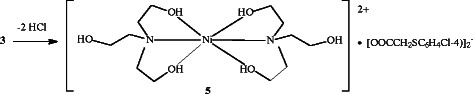
Conversion of compound 3 into compound 5.

Unlike compound **2**, compound **3** forms crystals **5** (Scheme 3).

**Figure 1:**
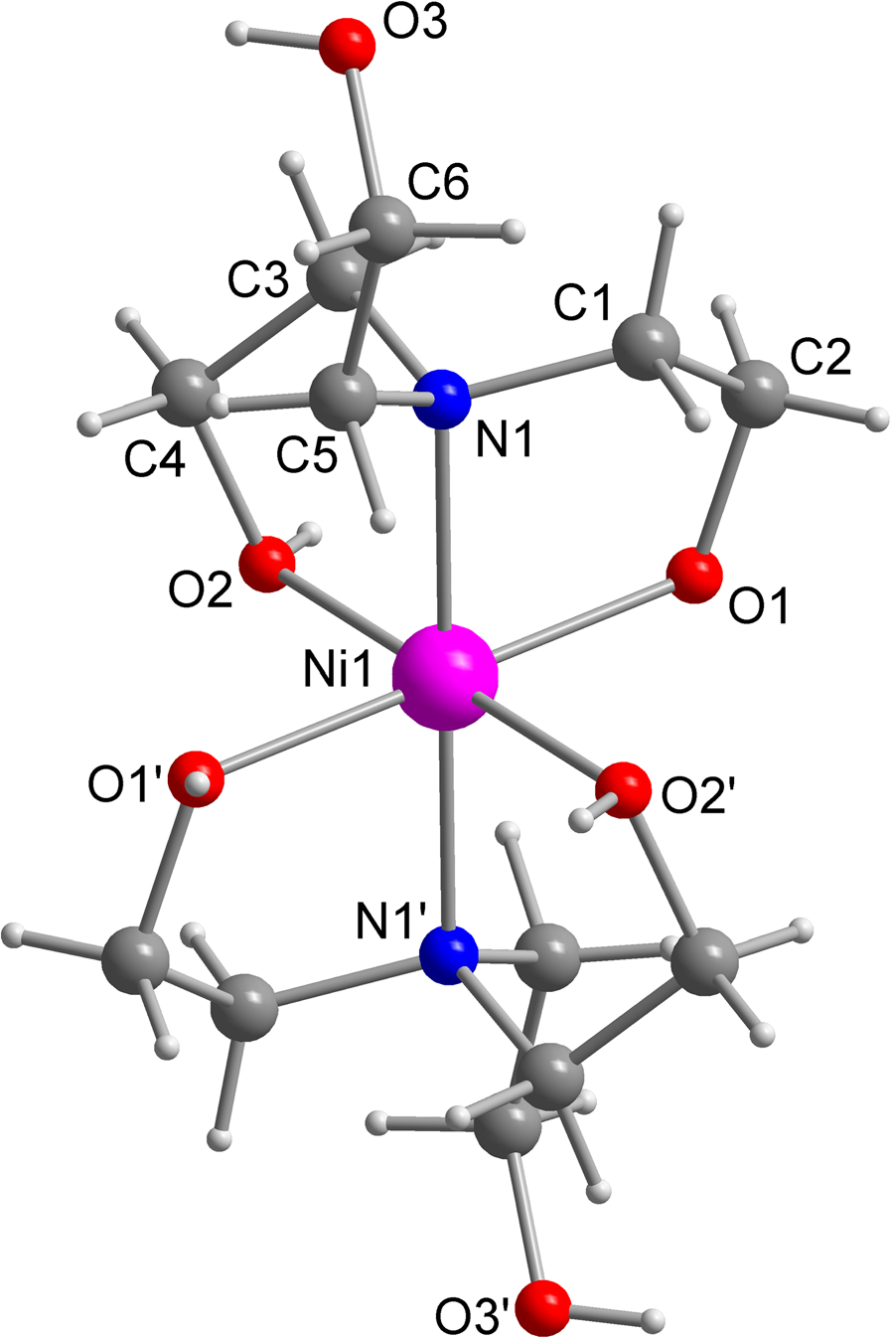
**Molecular structure of 5, showing the atom labelling.** The bis-[tris-(2-hydroxyethyl-amine)]-nickel(II) cation is centrosymmetric.

**Figure 2:**
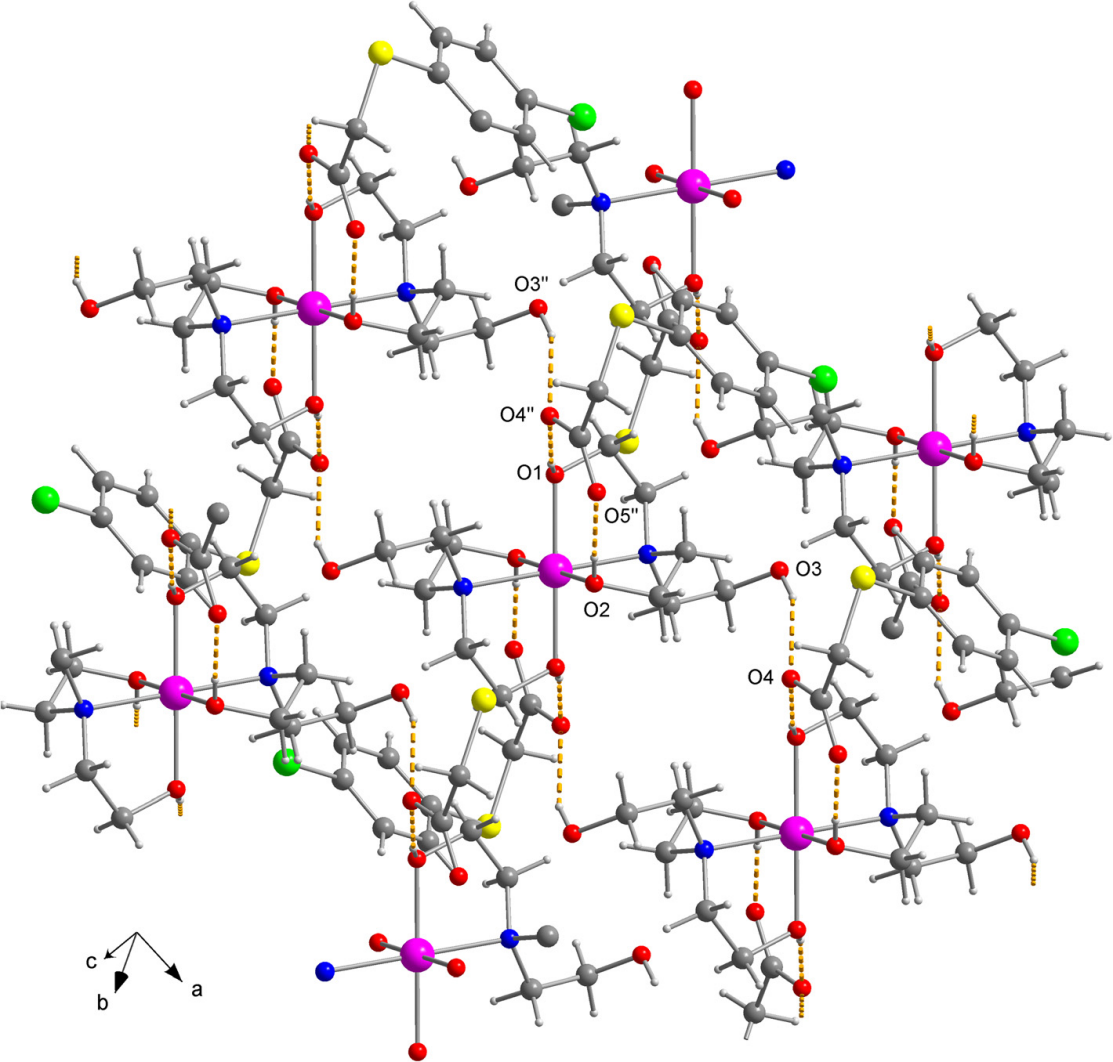
**Crystal packing of 5, illustrating the hydrogen bonds (dashed lines).** Hydrogen bonds geometry: O1∙∙∙O4'' 2.583(2) Å, H1∙∙∙O4'' 2.00(3) Å, O1-H1∙∙∙O4'' 171(5)°; O2∙∙∙O5'' 2.563(2) Å, H2∙∙∙O5'' 1.87(3) Å, O2-H2∙∙∙O5'' 177(3)°; O3∙∙∙O4 2.886(3) Å, H3∙∙∙O4 2.13(3) Å, O3-H3∙∙∙O4 158(3)°. Symmetry operator: '' x-1, y, z.

The structure of compound **5** was established by X-ray crystal structure analysis. The molecular structure with the atom labeling scheme is given in Figure  1. The packing diagram is shown in Figure 2.

The Ni(II) cation is coordinated by four oxygen atoms of the hydroxyl groups and two nitrogen atoms forming an weakly distorted octahedral coordination environment. The asymmetric unit contains only the half of the cationic moiety. Because nickel is located on an inversion centre the second half is generated by inversion. The nitrogen atoms occupy the *trans* positions of the coordination polyhedron. Resulting from symmetry, the Ni-N bond lengths are equal being 2.097(2) Å, and the N-Ni-N bond angle is 180°. The Ni-O distances are 2.062(2) Å and 2.070(2) Å. One hydroxyl group of each ethanol substituent is not involved in the coordination and directed away from the coordination centre. The N-Ni-O bond angles range from 81.50(7)° to 98.50(7)° and the O-Ni-O bond angles between 85.12(7)° and 94.88(7)°. Previously, structures containing the bis(triethanolamine)nickel (II) cation were described [25,26]. The 4-chloro-phenylsulfanyl unit is planar. The acetate substituent and the phenyl ring are almost in a tetrahedral arrangement with a C-S-C angle of 101.4(1)°. The carboxylic group is rotated around the S-C bond characterized by a C-S-C-C torson angle of −50.7(3)°. Cations and anions are linked by hydrogen bonds (see Figure  2). All oxygen atoms of the OH groups of tris-(2-hydroxy-ethyl)amine are involved in hydrogen bonds. Strong hydrogen bonds can be observed between those oxygen atoms, which are coordinated to the metal centre and both oxygen atoms of one carboxylic moiety. More weak hydrogen bonds are formed between the non-coordinated peripheric oxygen atoms of the OH groups and one oxygen atom of a carboxylic group. One oxygen atom (O4) of the carboxylic group forms bifurcurated hydrogen bonds, one to the coordinated oxygen atom O3 and another one to the noncoordinated oxygen atom O3. That leads to a three-dimensional polymeric network.

### Experimental

IR spectra (ν, cm^–1^) were recorded on a Varian 3100 FT- IR75 spectrophotometer (KBr). NMR spectra (δ, ppm) were measured on a DPX 400 instrument at 25°С. Reflections were collected using a STOE Imaging Plate Diffraction System (IPDS-II) at 210 K. The data were corrected for Lorentz, polarisation and extinction effects. No absorption correction was applied. The structure was solved by direct methods as implemented in the program SHELXS-97 [27]. The refinement was carried out using SHELXL-97 [28]. All the non-hydrogen atoms were refined anisotropically. The hydrogen atoms of the phenyl groups were calculated in their expected positions. All the other hydrogen bonds were located from the difference Fourier map. The hydrogen atoms were refined isotropically. For the phenyl and methylene hydrogen atoms a riding model was used. The other hydrogen atoms were free refined. For the visualisation of the structure the program DIAMOND [29] was applied. CIF data: Additional file 1. CCDC reference number: 876072.

### Synthesis

*Tris-(2-hydroxyethyl)ammonium 4-chlorophenylsulfanylacetate (****1***) [30] was synthesized in the following manner. To a solution of 4-chlorophenylsulfanylacetic acid 4-Cl-C_6_H_4_SCH_2_COOH (20.25 g, 0.1 mol) in MeOH (100 ml), was added dropwise a methanol (50 ml) solution of tris-(2-hydroxyethyl)amine (14.92 g, 0.1 mol). The mixture was stirred at 25°C for 30 min. The solvent was distilled in vacuum. The solid residue was repeatedly washed with ether and dried in vacuum to afford colorless powder (34.64 g, 98.5% yield), m.p. 90–92°C. For analytical characterization - see [30].

*Compound (****2****):* To the solution of 7.03 g (0.02 mol) of **1 (**m.p. 91°С) in 20 ml of МеOН the solution of 1.63 g (0.01 mol) ZnCl_2_ · 1.5Н_2_O in 5 ml of МеOН was added dropwise. The reaction mixture was stirred at 25°С for 12 h, the solvent was removed in a vacuum. The solid residue was thoroughly washed with ether and dried over Р_2_O_5_ in a vacuum. **2** (3.71 g, 43%) of colorless powder was obtained, m.p. 152°С, readily soluble in alcohols and moderately in H_2_O. IR: 1439 ν_s_(COO), 1553 ν_as_(COO), 3305 (ОН). ^1^Н NMR (100 MHz, *d*_*4*_-methanol): 7.30 -7.23 (4H, m, С_6_Н_4_), 3.84 (6H, t, OСН_2_), 3.63 (2H br s, SСН_2_), 3.28 (6H, t, NСН_2_). ^13^С NMR (400 MHz, *d*_*4*_-methanol): 174.82 (С = O), 135.84-128.2 (С_6_Н_4_), 55.52 (OСН_2_), 55.20 (NСН_2_), 37.37 (SСН_2_). Anal. Calc. for С_28_Н_44_O_10_S_2_N_2_Cl_4_Zn: С 40.00; Н 5.24; Cl 16.90; S 7.62; Zn. 7.78. Found: С 39.67; Н 5.78; Сl 16.97; S 8.26; Zn 7.82.

*Compound****(3)***: To the solution of **1** (0.703 g, 0.002 mol) in methanol (10 ml) was added dropwise a methanol solution (10 ml) of NiCl_2_ · 6H_2_O (0.237 g, 0.001 mol). The reaction mixture was stirred at 25°С for 15 h. The solvent was distilled in vacuum to give light-green powder **3**, m.p. 170°С. Yield 0.55 g (59%). Well soluble in Н_2_О, less soluble in alcohols. IR: 1401 ν_s_(COO), 1583 ν_as_(COO), 3312 (ОН). ^1^Н NMR (100 MHz, *d*_*4*_-methanol): 7.33-7.01 (4Н, m, С_6_Н_4_S), 3.91 (6Н, t, ОСН_2_), 3.65 (2Н, c, SСН_2_), 3.44 (6Н, t, NСН_2_). ^13^С NMR (400 MHz, *d*_*4*_-methanol): 177.09 (С = О), 131.76 –128.25 (С_6_Н_4_S), 55.33 (ОСН_2_), 54.98 (NСН_2_), 37.69 (SСН_2_). Anal. Calc. for С_28_Н_44_Cl_4_N_2_О_10_S_2_Ni: C 40.30; H 5.27; Cl 17.01; Ni 7.04. Found: C 41.13; H 4.99; Cl 17.29; Ni 6.88.

*Zinc di-(4-chlorphenylsulfanyl)acetate dihydrate****(4)***: 0.5 g of **2** was dissolved in 10 ml of aqueous alcohol upon stirring (75°C), the solution was kept for one month at room temperature and filtered. The solid residue was washed with ether and dried over Р_2_O_5_ to obtain colorless plate crystals **4** with m.p. 202°С. IR: 1416 ν_s_(COO), 1540 ν_as_(COO), 3240 (ОН). ^1^Н NMR (100 MHz, *d*_*4*_-methanol): 7.31-7.21 (4H, m, С_6_Н_4_), 3.63 (2H, br.s, SСН_2_). ^13^С NMR (400 MHz, *d*_*4*_-methanol): 175.02 (С = O), 131.80-128.65 (С_6_Н_4_), 37.37 (SСН_2_). Anal. Calc. for С_16_Н_16_O_6_S_2_Cl_2_Zn: С 38.01; Н 3.17; Zn 12.95. Found: С 38.17; Н 3.18; Zn 12.82.

*Bis-[(tris-2-hydroxyethyl)ammonium]nickel(II) di-(4-chlorphenylsulfanyl)acetate****(5)***: From a solution **3** (aqueous alcohol, 75°C), the blue crystals **5** were obtained (20°C, for one month), m.p. 176^о^ С. ^1^Н NMR (100 MHz, *d*_*4*_-methanol): 7.13-6.79 (4Н, m, С_6_Н_4_S), 3.49 (6Н, t, ОСН_2_), 3.29 (2Н, c, SСН_2_), 2.67 (6Н, t, NСН_2_). ^13^С NMR (400 MHz, *d*_*4*_-methanol): 178.90 (С = О), 136.16 -128.40 (С_6_Н_4_S), 56.50 (ОСН_2_), 54.43 (NСН_2_), 37.62 (SСН_2_). Anal. Calc. for C_28_H_42_Cl_2_N_2_O_10_S_2_Ni: C 44.18; H 5.79; Cl 9.33; Ni 7.72. Found: С 43.88; Н 5.63; Cl 9.54; Ni, 8.01.

#### Crystal data of 5

*C*_*28*_*H*_*42*_*Cl*_*2*_*N*_*2*_*NiO*_*10*_*S*_*2*_*, M = 760.37, triclinic, a = 8.0020(8), b = 9.4980(10), c = 11.6401(12) Å, V = 823.29(15) Å*^*3*^*, T = 210(2) K, space group P-1 (no.2), Z = 1, μ(MoKα) = 0.936 mm*^*-1*^*; 5252 reflections measured, 2714 unique (R*_*int*_ *= 0.031) which were used in all calculations. Final R values: wR*_*2*_*(F*^*2*^*) = 0.0603, R*_*1*_ *= 0.0445 (all data); wR*_*2*_*(F*^*2*^*) = 0.0575, R*_*1*_ *= 0.0300 [I > 2σ)].*

## Conclusion

The reaction of pharmacological active ionic liquid H^+^N(CH_2_CH_2_OH)_3_ ∙ (^-^OOCCH_2_SC_6_H_4_Cl-4) **(1)** with zinc or nickel chloride affords stable at room temperature powder-like adducts [H^+^N(CH_2_CH_2_OH)_3_]_2_ ∙ [M(OOCCH_2_SC_6_H_4_Cl-4)_2_Cl_2_]^2-^, M = Zn **(2)**, Ni **(3)**. By recrystallization compound **2** unexpectedly gives Zn(OOCCH_2_SC_6_H_4_Cl-4)_2_ ∙ 2H_2_O **(4)**. Unlike **2**, compound **3** gives crystals [N(CH_2_CH_2_OH)_3_]_2_Ni^2+^ · [^-^OOCCH_2_SC_6_H_4_Cl-4]_2_**(5)**, which have a structure of metallated ionic liquid. It is the first example of the conversion of a protic ionic liquid into metallated ionic liquid (compound **1** → **3** → **5**). The structure of **5** has been proved by X-ray diffraction analysis. The investigation of physiological activity of metallated ionic liquids will be conducted in a new future.

## Competing interests

The authors declare that they have no competing interests.

## Authors’ contributions

SNA carried out the synthetic experiments and drafted the manuscript. ANM has formulated the research idea and prepared the manuscript draft version. RGM prepared the manuscript for submission and coordinated final formulation. US collected the X-ray data and performed the structure solution. All authors read and approved the final manuscript.

## Supplementary Material

Additional file 1 Crystallographic information. Contains all relevant CIF information. Click here for file
